# Heterogeneous Suppression of Sequential Effects in Random Sequence Generation, but Not in Operant Learning

**DOI:** 10.1371/journal.pone.0157643

**Published:** 2016-08-18

**Authors:** Hanan Shteingart, Yonatan Loewenstein

**Affiliations:** 1 The Edmond & Lily Safra Center for Brain Sciences, The Hebrew University of Jerusalem, Jerusalem, Israel; 2 Department of Neurobiology, The Alexander Silberman Institute of Life Sciences and the Federmann Center for the Study of Rationality, The Hebrew University of Jerusalem, Jerusalem, Israel; Universidad de Alicante, ITALY

## Abstract

There is a long history of experiments in which participants are instructed to generate a long sequence of binary random numbers. The scope of this line of research has shifted over the years from identifying the basic psychological principles and/or the heuristics that lead to deviations from randomness, to one of predicting future choices. In this paper, we used generalized linear regression and the framework of Reinforcement Learning in order to address both points. In particular, we used logistic regression analysis in order to characterize the temporal sequence of participants’ choices. Surprisingly, a population analysis indicated that the contribution of the most recent trial has only a weak effect on behavior, compared to more preceding trials, a result that seems irreconcilable with standard sequential effects that decay monotonously with the delay. However, when considering each participant separately, we found that the magnitudes of the sequential effect are a monotonous decreasing function of the delay, yet these individual sequential effects are largely averaged out in a population analysis because of heterogeneity. The substantial behavioral heterogeneity in this task is further demonstrated quantitatively by considering the predictive power of the model. We show that a heterogeneous model of sequential dependencies captures the structure available in random sequence generation. Finally, we show that the results of the logistic regression analysis can be interpreted in the framework of reinforcement learning, allowing us to compare the sequential effects in the random sequence generation task to those in an operant learning task. We show that in contrast to the random sequence generation task, sequential effects in operant learning are far more homogenous across the population. These results suggest that in the random sequence generation task, different participants adopt different cognitive strategies to suppress sequential dependencies when generating the “random” sequences.

## Introduction

### The Unpredictability of Behavior

In every-day experience, some aspects of human and animal behaviors seem unpredictable. Similarly, in laboratory settings, we are rarely able to fully predict behavior. For example, when humans are instructed to repeatedly generate the same hand trajectory, substantial trial-to-trial variability in the hand trajectory is observed [1]. Another example is in perceptual tasks, where there is a trial-by-trial variability in the response to the same stimuli [2]. Yet another example is that when repeatedly instructed to choose from the same set of options, human choices are often inconsistent, a phenomenon termed *stochastic choice* [3]. Indeed, when modeling behavior, it is standard to incorporate a stochastic term to account for the unexplained variance. While this unpredictability is typically referred to as *stochastic* and is regarded as a nuisance *noise*, it may also serve a function, e.g. in strategic settings [4] or to enable exploration [5]. Stochastic behavior is also, in some cases, the only self-consistent behavior in Partially Observable Markov Decision Processes [6]. Supporting the functional role of stochasticity are several studies demonstrated that the level of unpredictability can be learned (for a review see [7]).

### The Predictability of ‘Random’ Behavior

There is a long tradition of experiments, in which human participants are explicitly instructed to generate or evaluate random sequences of discrete symbols, e.g. *Heads* (denoted as *H* or 1) and *Tails* (denoted as *T* or 0) [8]. These studies report systematic deviations from randomness [4,9–20], as demonstrated in more details in the Results section. Previous studies have shown that central executive component of working memory are involved in the task of random sequences generation (RSG) [20,21]. As a supportive evidence, consider that frontal lobe lesions, neurodegeneration and other diseases affecting the central nervous system result in impairments in this task (see [22] for review).

Over the years, there have been several attempts to model deviations from randomness in RSG tasks [12]. The *alternation bias*, namely, the tendency to alternate more than expected by chance, has received a lot of attention and was quantified using a first-order Markov model [23]. However, by construction, this model was unable to account for higher-order statistics observed in behavior (see also below). Similarly, the *local representativeness* hypothesis [24] focuses on the first moment, computed over a small number of trials in RSG, and tends to disregard the higher order moments of the statistics, e.g. the effect on the next coin generation of past trials beyond the recent one. A quantitative version of this model was indeed able to explain some of the observed deviations from randomness, however, the model’s assumptions were inconsistent with the behavior of a substantial fraction of the participants [4]. Modern approaches focused on predicting of next choice in the sequence using Machine Learning methods. For example, in one study, a pattern-based approach was used to train a predictor of the next choice by minimizing a non-linear distance function [22]. In another study, a linear-support-vector-machines (SVM) was used for prediction [25]. While these models were partially successful in predicting behavior, the cognitive processes underlying deviations from randomness patterns remain elusive.

While some deviations from randomness are evident when averaging over the different participants, there are also substantial differences between participants. For example, one study has reported that 71% of the participants exhibited the alternation bias described above, while a minority of participants exhibited an opposite bias–an *inertia bias* [23]. Moreover, taking participants’ heterogeneity into account was shown to increase the predictive power of a pattern-based model [22,25]. These results suggest that different participants employ different cognitive strategies in RSG tasks. In this study we quantify this heterogeneity and compare it to the one observed in Operant Learning.

### Sequential Effects in Behavior

It is well established that in sequential tasks, participants’ behavior depends upon previous trials in a systematic manner [26]. For example, in perception, such sequential effects may reflect statistical learning [27]. The alternation and inertia biases described above are merely particular examples of first-order sequential effects: the response of the participant is biased by her response in the preceding trial.

In order to quantify sequential effects in the RSG task, we use the logistic regression model [28]. According to this model, the probability of choosing an action *a* at time *t* (*a*_*t*_), assuming there are only two available actions, *a* and $\bar{a}$, is given by:

$$\mathrm{Pr}\left(a_{t}\right)=\frac{1}{1+\exp(-\Delta Q_{t})}$$ (1)

where Δ*Q*_*t*_ is a linear function of past actions, such that

$$\Delta Q_{t}=\sum_{k=1}^{L}\beta_{k}\left(a_{t-k}-{\bar{a}}_{t-k}\right)+\beta_{0}$$ (2)

where *a*_*t*–*k*_ ∈ {0,1} and ${\bar{a}}_{t-k}\in \{0,1\}$ are index variables that denote the history of past choices, *β*_*k*_ are parameters and *L* is the model horizon (also known as order or duration in terms of trials).

To gain insight into Eq (2), we note that random, unbiased sequence emerges if all parameters vanish i.e. *β*_*i*_ = 0. A general preference in favor of *a* will manifest as *β*_0_ > 0. Finally, the value of *β*_1_ will determine whether the subject has a propensity to alternate (*β*_1_ < 0) or whether she is biased towards inertia (*β*_1_ > 0).

### Contribution of this Study

Utilizing logistic regression to analyze behavior in the RSG task allows us to go beyond previous studies in quantifying deviations from randomness in this task. In particular, this framework allows us to quantify sequential effects, study their stationarity and their heterogeneity between participants. We find that deviations from randomness are primarily due to sequential effects at a time scale of several trials and that these deviations are stable over hundreds of trials. Moreover, heterogeneity between participants dominates sequential effects in this task. We further show that the logistic regression framework is a reminiscence of the valuation system framework in operant learning. This allows us to compare the level of heterogeneity in the sequential effects in the two tasks. We find that (reward-independent) sequential effects in operant learning are larger and more homogeneous than sequential effects in the RSG task. These results suggest that the RSG task is associated with the suppression of sequential effects, and that different participants use different cognitive strategies to suppress these sequential effects.

## Results

### Sequential Effects of Random Choice

30 participants were instructed to generate a sequence of 1,000 random binary numbers 1 and 0, representing *H*, and *T*, respectively; [15], see also Materials and Methods. In order to quantify sequential effects of random number generation in a model-free fashion, we computed the conditional probabilities Pr(*a*_*t*_ = 1|*a*_*t*–*k*_ = 1) of generating *H* in a current trial (*a*_*t*_ = 1), conditioned on action *H k* trials ago (*a*_*t*–*k*_ = 1). Note that for a sequence generated by independent tosses of an unbiased coin, all conditional probabilities are equal to 0.5, Pr(*a*_*t*_ = 1|*a*_*t*–*k*_ = 1) = 0.5.

**Fig 1:**
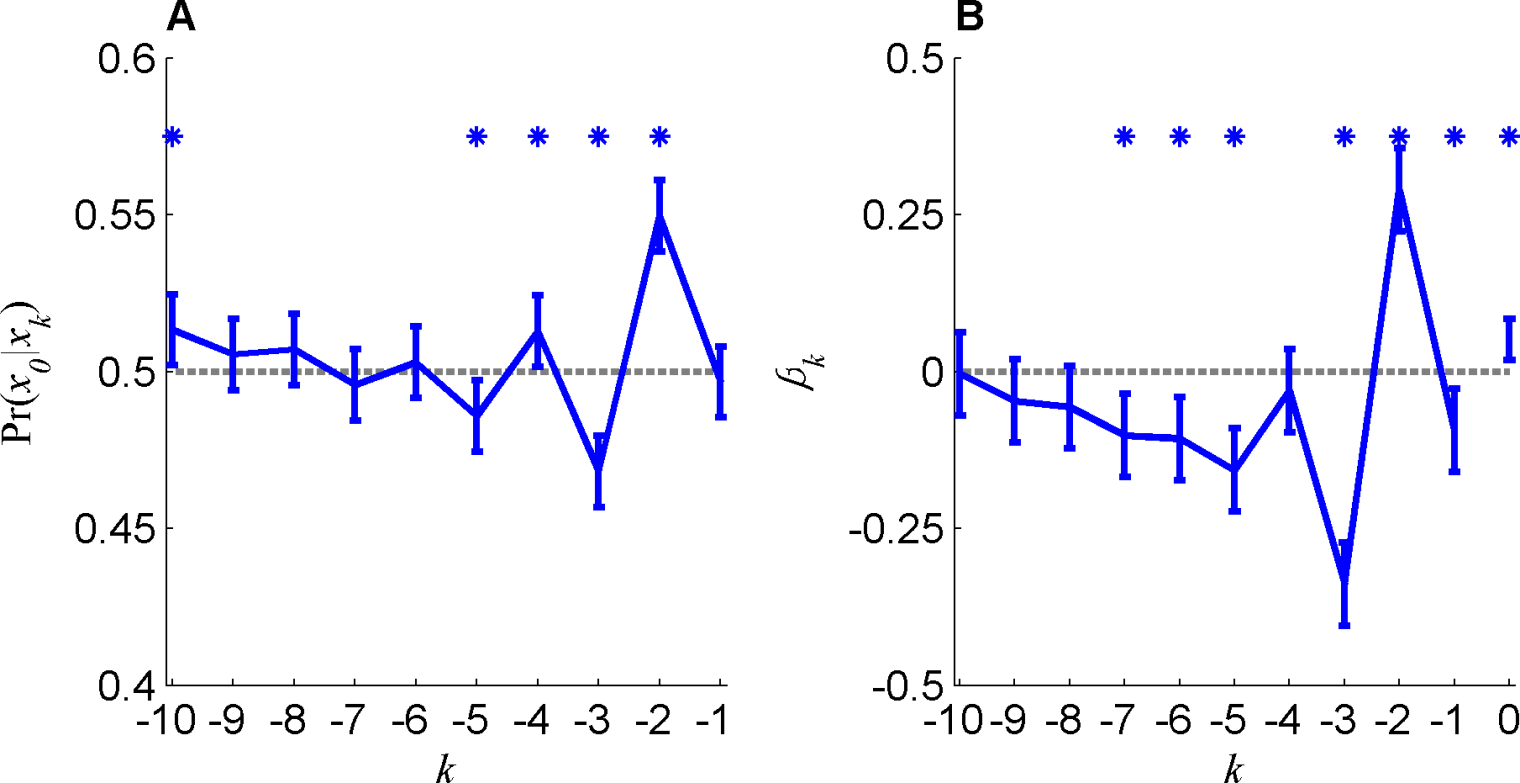
Sequential effects in random sequence generation task. A. The conditional probabilities Pr(*x*_0_ = *H*|*x*_*k*_ = *H*) of generating *H* in a current trial (*x*_0_ = *H*), conditioned on a choice *H*–*k* trials ago. B. Logistic regression model's coefficients. Dashed gray line represents the null hypothesis of no sequential effect. Error bars represents the confidence interval at significance level of 0.005 (Bonferroni correction for multiple comparison) and stars denoted statistical significance assuming normal distribution of noise (t-test).

By contrast, averaging over all trials, we found, as depicted in Fig 1A, significant deviations of the conditional probabilities from 0.5 (for *k* = 2,3,4,5,10, P < 0.001).

In typical sequential effects, the influence of a trial on subsequent trials decays with the number of interleaving trials e.g. in perception [27] and in operant learning (OL) [29]. By contrast, in the RSG task, the most recent trial (*k* = 1) seemed to have little effect on the current trial, whereas more preceding trials seemed to have a larger effect on behavior. This seeming contradiction is addressed in the next section.

In Fig 1A we considered the influence of each past trial separately. In order to study the joint influence of the sequence of past trials, we used the logistic regression model (Eqs 1 and 2). Fig 1B (blue) depicts the regression coefficients that maximize the likelihood of the observed sequence given this model. These coefficients are qualitatively similar to the conditional probabilities depicted in Fig 1A and significantly different from zero (for *k* = 0,1,2,3,5,6,7, P < 0.001).

### Heterogeneity of Sequential Effects

**Fig 2:**
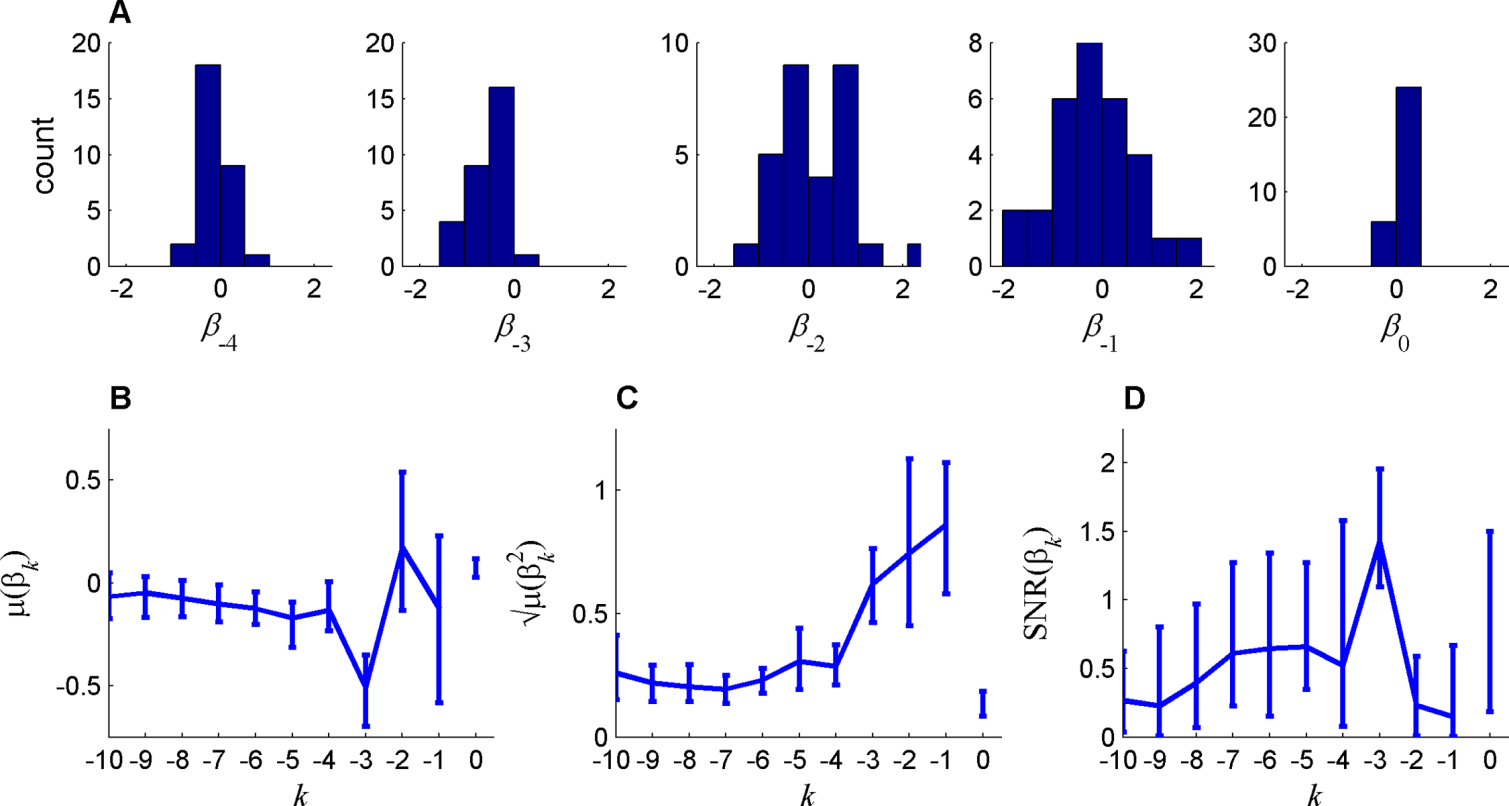
Heterogeneity of sequential effects in random sequence generation task, quantified using the coefficients of a logistic regression of memory size *L* = 10. A. The distribution of the coefficients across participants for the bias and lags 1–4 coefficients. The statistics of these coefficients are summarized in B, C and D for the mean (*μ*), magnitude ($\sqrt{\mu \left(\beta_{k}^{2}\right)}$) and the signal to noise ratio (SNR), respectively, as a function of the lag (*k*), where the bias terms (*k* = 0) is at the right of *k* = -1). Error bars represents the 95% confidence intervals computed by 100-fold bootstrapping (Bonferroni corrected for multiple comparison).

The conditional probabilities and the regression coefficients depicted in Fig 1A and 1B, respectively, were based on a population analysis and thus ignored possible individual differences between the participants. In order to study these individual differences, we estimated the regression coefficients for each participant separately. The results of this analysis are presented in Fig 2A, where we plot the distribution of the first five regression coefficients, (*β*_*k*_, *k* = 0,…,4) over the population of participants. The averages of these coefficients across the participants *μ*(*β*_*k*_), which correspond to the common or the homogeneous components of the sequential effect, are qualitatively similar to the regression coefficients computed using the pooled data (compare Fig 2B to Fig 1B). In particular, the average coefficients analysis implies that the most recent trial has a relatively small effect on behavior (*μ*(*β*_1_) = -0.09 (CI = [-.45, .2], P > .4)). However, when considering the regression coefficients of each participant separately (Fig 2A), we find that the value *β*_1_ of most participants was significantly and substantially different from zero (for 20 out of 30 of the participants P < 0.05/30, where the division by 30 corresponds to Bonferroni correction for multiple comparison). It was positive for some participants and negative for others, such that the magnitude of the average, *μ*(*β*_1_) is small.

To further measure individual sequential effects, we quantified the magnitude of the regression coefficients by computing the quadratic mean of the coefficients ($\sqrt{\mu \left(\beta_{k}^{2}\right)}$). As depicted in Fig 2C, the magnitude of the coefficients decays with the number of interleaving trials, in line with standard sequential effects in other fields of psychology. Thus, the atypical (non-monotonous) regression coefficients depicted in Fig 1 and Fig 2B are due to substantial heterogeneity in sequential effects between participants.

The relative contribution of participants’ coefficients heterogeneity to their behavior can be further quantified by computing, for each coefficient, the signal to noise ratio (SNR): the ratio of the absolute mean |*μ*(*β*_*k*_)| to the standard deviation *σ*(*β*_*k*_) across the population. The SNR is a non-negative statistic where the larger the value of the SNR, the more similar is the temporal dependency of participants in sequence generation. As depicted in Fig 2C, heterogeneity is maximal for the first two coefficients and is substantially lower for longer intervals. This is particularly true for lag 3, where participants were unanimously biased in favor of choosing a symbol that is opposite to that of three trials ago. In summary, we find substantial sequential effects in the RSG that decay with the temporal lag. For short lags, associated with large magnitude coefficients, sequential effects are dominated by participants’ heterogeneity whereas for long lags, associated with small magnitude coefficients, participants are substantially more similar.

### Reproducing Deviations from Randomness

**Fig 3:**
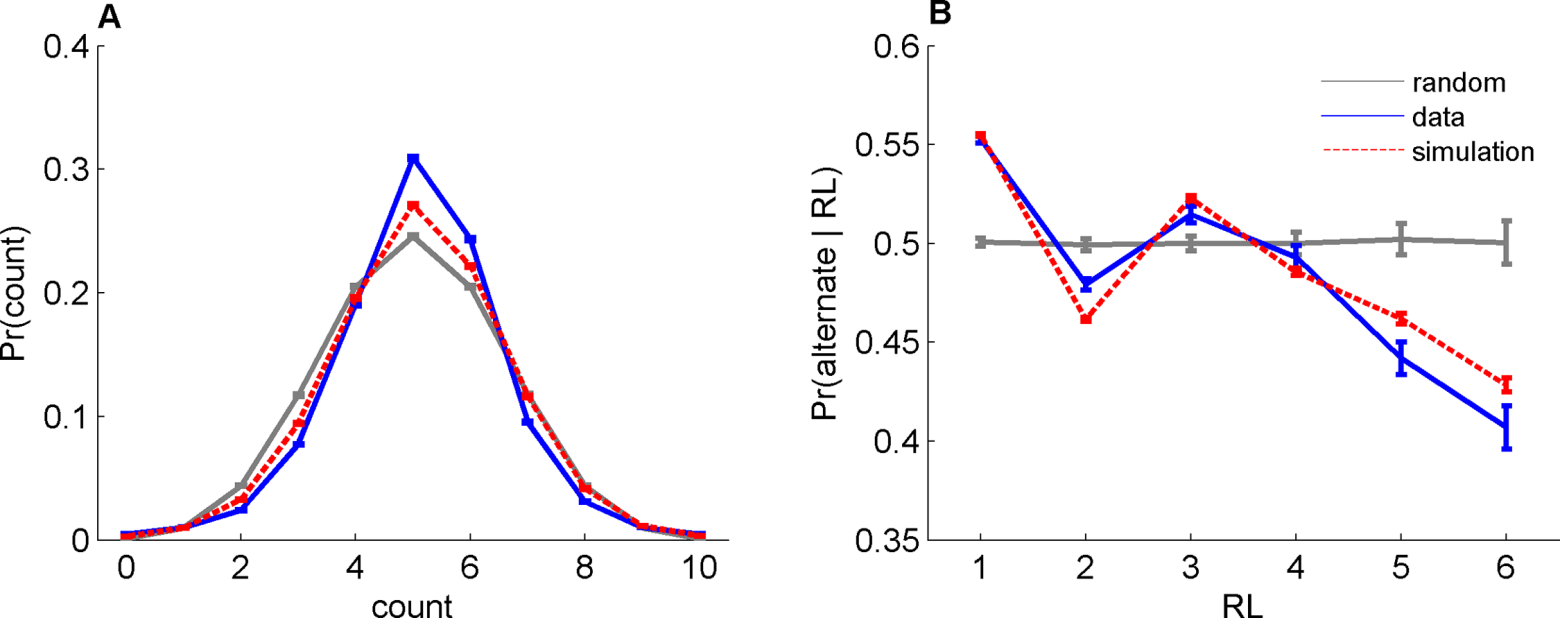
Deviations from randomness (gray) in behavior (blue) and logistic model (red). A. The distribution of Heads’ counts in ten consecutive trials. B. The probability to alternate after a RL trials devoid of alternations (strike). Error bars are SEM.

To test the logistic model consistency with previously-reported deviations from randomness, we used the individual participant regression coefficients (computing then up to a maximum lag of *L* = 3) to simulate synthetic sequences per participant. We compared the results of the simulation with the empirical data in view of two previously-reported common deviations from randomness [15]. First, previous studies have reported that people tend to generate sequences that are more balanced (equal fraction of Heads and Tails) than expected by chance. This is depicted in Fig 3A, where we plot the probability of number of Heads in a sequence of length 10. In line with previous studies, the histogram of the participants’ behavior (blue) is narrower than expected by chance (gray, P < 10^−74^_,_ t-test on Monte-Carlo simulation) and the bin at 5 (balanced sequence) is higher than expected by chance (P < 10^−144^). Similar results, namely narrower histogram and a larger fraction of balanced sequences than expected by chance is also observed in the simulated sequences (red dots, P < 10^−3^ and P < 10^−24^, respectively). Moreover, it is well-known that participants tend to alternate (act differently from last action) more than expected by chance. One way of quantifying this effect is to consider the probability of alternation as a function of the preceding *running lengths* (RL), which is the length of the preceding sequence that is devoid of alternations. This is depicted in Fig 3B, which shows that indeed participants are more likely to alternate than expected by chance after a 1-trial RL (compare blue to gray, P < 10^−33^). However, they tend to alternate less than predicted by chance for long RLs, a phenomenon which can be regarded as an “anti-gambler fallacy” [15]. Similar to the experimental data, the model (red) can account for these anomalies qualitatively. Yet, there are quantitative differences which may be the result of the limitation of the logistic model, as discussed below.

### Testing the Model Predictive Power

**Fig 4:**
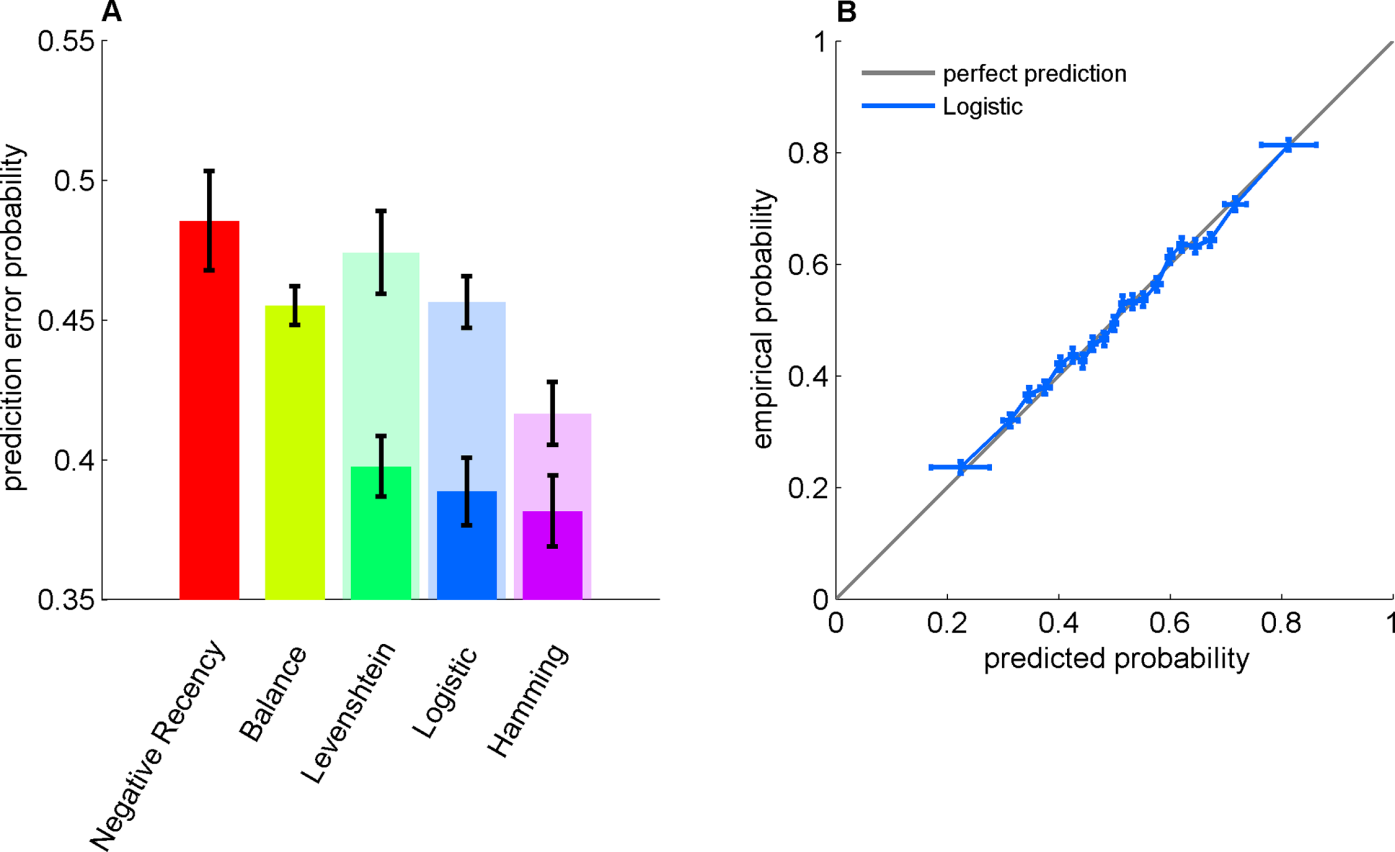
Predicting choices in the RSG task. A. Prediction-error probability for different models: Negative Recency (red), Balance (mustard), Levenshtein (green), Logistic (blue) and Hamming NN (purple). Dark and light colors correspond to the heterogeneous versions (a different set of parameters for each participant) and homogeneous versions (a single set of parameters for all participants) of the models, respectively. The model memory lengths (*L*) were chosen to minimize the generalization error: Negative Recency, *L* = 1; Balance, *L* = 7; Heterogeneous Levenshtein, *L* = 3; Homogeneous Levenshtein, *L* = 4; Heterogeneous Logistic, *L* = 3; Homogeneous Logistic, *L* = 8; Heterogeneous NN, *L* = 4; Homogeneous NN, *L* = 8. B., The predicted probability for Heads versus the empirical one (in the test set) for the logistic model is depicted in panel B (blue) over the diagonal (gray). B. Empirical probability of *H* as a function the predicted probability of *H*. The data was divided into 20 equally populated bins according to the predicted probability of *H* and the fraction of *H* was computed for each bin. Error bars represent the SEM.

The results presented in the previous section (Figs 1 and 2) indicate that sequential effects, modeled using the logistic-regression model, can qualitatively account for some of the deviations of the generated sequences from randomness (Fig 3). Moreover, this model also allows us to *quantitatively* test the fraction of regularity in the human behavior that is captured by these previously-reported deviations from regularity, as well as to *qualitatively* estimate the extent to which regularities in the RSG are subject-specific. To that goal, we compared the predictive power of alternative homogeneous and heterogeneous models that predict the current trial based on the preceding *L* trials. For each model, we computed the prediction error (see Materials and Methods) as a function of the model memory length (characterized by the maximum lag coefficient *L*) and considered the minimal prediction error over the different values of *L* as a measure of the performance of the model (Fig 4A).

We first considered two parameter-free baseline models, which are based on the previously-described regularities in RSG. First, the *negative-recency* model (red, *L* = 1) predicts alternation from previous trial. We found that the predictive power of the negative-recency model is not-significantly different from chance (Fig 4A; .48, CI = [.45, .52], P = 0.2, t-test over participants), consistent with our finding that Pr(*a*_*t*_ = 1|*a*_*t*–*k*_ = 1) is not significantly different from 0.5 (Fig 1A). Next, we considered a *balance* model (mustard), in which the generated symbol is chosen as to balance the number of Heads and Tails in the local sequence, in line with the local representativeness hypothesis (see Introduction). We found that the balance model was slightly but significant better than chance (.46 CI = [.44, .47], P < 10^−6^).

In addition to the two parameter-free baseline models, we considered three models: (1) the logistic regression model discussed above (blue); (2) the recently-used pattern-matching *Levenshtein Distance model* [22] (green) and (3) a Hamming nearest-neighbor (NN) model (purple; Materials and Methods) that in the limit of an infinite number of samples is nearly optimal [30]. By comparing the predictive power of the logistic regression model to the Levenshtein and NN models, we can assess the extent that the specific logistic framework captures the regularities present in behavior in the RSG task.

The negative recency and balance models, which are typically considered when discussing regularity in the RSG task do not take into account individual differences. To study the role of these individual differences in predicting behavior, we considered a *homogeneous* version and a *heterogeneous* version of the models. In the former, a model was fitted to the entire population of participants, ignoring individual differences. In the latter, a model was fitted individually to each participant. Note that the homogeneous models ignore individual differences and therefore, with a large enough training set, they are sure do worse than their heterogeneous counterparts. However, this additional predictive potential comes at the expense of a larger sensitivity to parameters’ overfitting. Therefore, there is no theoretical guarantee that with a finite training set the predictive power of the heterogeneous models will be larger than that of the homogeneous models.

Considering the heterogeneous versions, the logistic regression model (blue) outperformed the Levenshtein Distance model (P = 0.006, pair-wise t-test over participants), with a prediction error of .39 (CI = [.36, .41], P < 10^−9^) and there was no significant difference between the performances of the logistic regression and the NN models (P = .1, CI = [0.35, 0.40]). These results indicate that the logistic regression model captures the regularities present in the behavior of the participants in the RSG task. Comparing the homogeneous and heterogeneous versions of the models we found that the predictive power of the homogeneous version of the three models (light colors) is significantly and substantially lower than that of the corresponding heterogeneous version (dark colors in Fig 4A, P < 10^−6^). In particular, while the prediction error of the homogeneous logistic regression model is 46%±1%, it is 39%±3% for the heterogeneous logistic regression model. In other words, by ignoring population heterogeneity we can account for less than half of the regularity in behavior in the task. These results emphasize the role of individual differences in regularities observed in the RSG task (note that the number of parameters is not and should not be controlled here because the out-of-sample predictive power, rather than the fit to the data, is compared between the different models).

As the logistic regression model is only a particular model in a larger family of generalized linear models, we tested whether the particular non-linearity used in the logistic regression is consistent with the data. To that goal, we computed the frequency of *H* as a function of the predicted probability of *H*. As depicted in Fig 4B, the observed probability is almost a linear function of the predicted probability (R = .99, P < 10^−17^), indicating that behavior is consistent with the particular non-linearity of the logistic regression model.

### Stationarity Analysis

An underlying assumption of the logistic model presented above (as well as the other models depicted in Fig 4) is stationarity, i.e., the parameters of the model do not change over time (trial number). To test this stationarity assumption, we studied whether better predictions can be achieved if the model parameters themselves are dynamic variables that slowly change over time. Specifically, we defined a *discounting parameter* that quantifies the weighting of past trials in the parameter estimation procedure and considered the discounting parameter that best predicts behavior (see Materials and Methods). The reciprocal of this discounting factor is a measure of the stationarity of the sequence. We found that the median and mean of the best-predictive reciprocal of the discounting factor over the participants was 500 and 2,000 trials, respectively (CI = [300, 1,000] and CI = [350, 5,400], respectively by bootstrapping 100 times). These results indicate that in the time-scale of the experiment (1,000 trials), the parameters that describe the behavior of the participants are relatively stable.

### Comparison to Operant Learning

The results presented in the preceding sections indicate that subject-to-subject heterogeneity dominates sequential effects in the RSG task. However, sequential effects are also present in other psychophysical tasks raising the question of whether heterogeneity dominates sequential effects also in these other tasks. To address this question, we quantified sequential effects in an operant learning (OL) task, in which participants repeatedly choose between two alternatives that provide monetary reward stochastically, according to their choices [31], see Materials and Methods).

It is generally believed that OL is achieved through the synergy of two processes. First, the *values* of different actions (or more generally, state-actions), which are a measure of their “attractiveness” are learned iteratively from past actions and their subsequent rewards. Second, these learned values are used to choose among the different actions such that actions associated with a higher value are more likely to be chosen again [32,33] (see [34] and [35], for review). Typically, a *softmax* function is used to model the mapping from values to actions [5] yielding Eq (1) where Δ*Q* denotes the normalized value of the two actions. While in machine-learning models the value of an action is simply a measure of the expected reward associated with that action, it is known that in human and animal behavior, reward-independent sequential effects also play a role in operant learning tasks [36–38]. Therefore, to model behavior in the operant learning task, we posit that choice probability is described by Eq (1), where

$$\Delta Q=\sum_{k=1}^{L}\left(a_{t-k}-{\bar{a}}_{t-k}\right)\left(\alpha_{k}r_{t-k}+\beta_{k}\right)+\beta_{0}$$ (3)

To gain insight to this equation we note that the first term in the second parenthesis in the right hand side of Eq (3) corresponds to the effect of the interaction of reward and action on behavior and therefore serves the traditional role of a “value”. In particular, if *β*_*l*_ = 0 (*l* ∈ {0,1,2,…,*L*}), $\alpha_{k}^{}=\frac{1}{T}\cdot \gamma^{k}$ where *T* > 0 and 0 < *γ* < 1 are parameters then in the limit of large *L* these equations simply describe the well-known Time Difference (TD) learning algorithm [5]. The second term of the right hand side of Eq (3) denotes sequential effects, as in Eq (2). Therefore, the parameters *β*_*l*_ correspond to the residual, reward-independent sequential effects. By comparing the population heterogeneity in *β*_*l*_ within the operant learning task with those parameters in the RSG task, we can study the extent to which the dominance of heterogeneity in the RSG task is a property of the task or a property of sequential effects in general.

**Fig 5:**
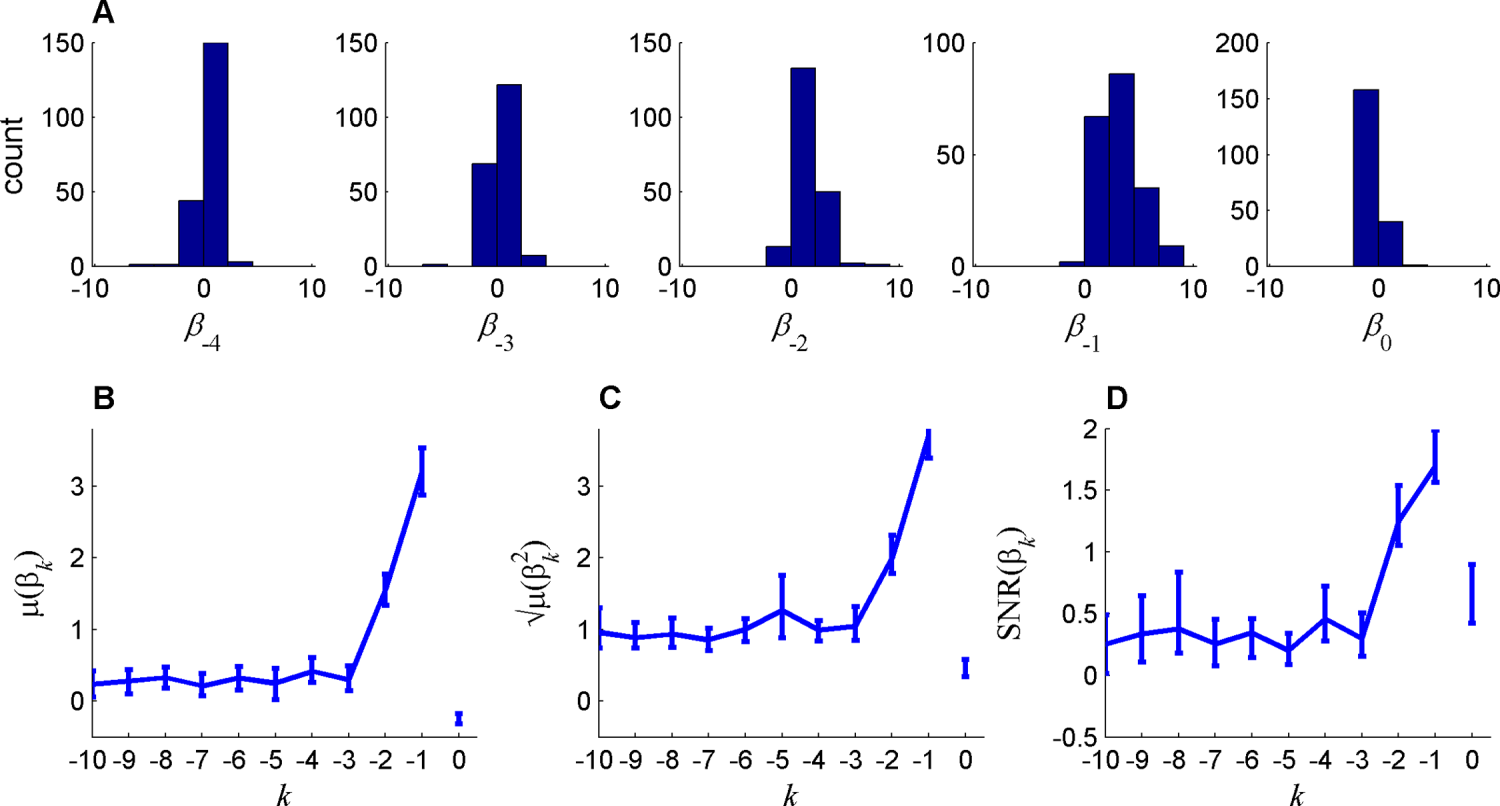
Heterogeneity of sequential effects in Operant Learning task quantified by the coefficients of a logistic regression of memory size *L* = 10. Panel A represents the distribution of the coefficients across participants for the bias and lags 1–4 coefficients. The statistics of these coefficients are summarized in Panel B, C and D for the mean (*μ*), standard deviation ($\sqrt{\mu \left(\beta_{k}^{2}\right)}$) and the signal to noise ratio (SNR), respectively. Error bars represents the 95% confidence intervals computed by 100-fold bootstrapping (Bonferroni corrected for multiple comparison).

For each participant, we estimated the parameters of Eq (3) and the results are depicted in Fig 5. Similar to the RSG task, there are significant residual sequential effects (Fig 5A), which decrease with the temporal lag (B and C). However, in contrast to the RSG task, these coefficients, averaged over the population, decrease monotonously with the lag, indicating that sequential effects in the operant learning are far more homogenous than in the RSG task. Considering the largest two terms, both in terms of their average value, *μ*(*β*_*k*_), and in terms of their quadratic mean $\sqrt{\mu \left(\beta_{k}^{2}\right)}$, *β*_1_ and *β*_2_, we find that these coefficients are substantially and significantly larger in the operant task than in the RSG task (*μ*(*β*_1_)^*OL*^ – *μ*(*β*_1_)^*RSG*^ = 3.3, *CI* = [2.6,4], *P* ∼ 0, *μ*(*β*_2_)^*OL*^ – *μ*(*β*_2_)^*RSG*^ = 1.4, *CI* = [.9,1.8], *P* < 10^−7^ $\sqrt{\mu {\left(\beta_{1}{}^{2}\right)}^{OL}}-\sqrt{\mu {\left(\beta_{1}{}^{2}\right)}^{RSG}}=3.6,CI=[2.7,4.6],P<10^{-5}$ and $\sqrt{\mu {\left(\beta_{2}{}^{2}\right)}^{OL}}-\sqrt{\mu {\left(\beta_{2}{}^{2}\right)}^{RSG}}=1.8,CI=[1.1,2.4],P=0.003$).

Nonetheless, the SNR of these coefficients in the OL task is far smaller than that in the RSG task ($SNR_{1}^{OL}=0.15,CI=[0-0.5];\;SNR_{1}^{RSG}=1.7,CI=[1.5-1.8],P∼0$ and $SNR_{2}^{OL}=0.2,CI=[0-0.6];\;SNR_{2}^{RSG}=1.2,CI=[1,1.4],P∼0$). These results indicate that the substantial heterogeneity observed in the values of the first two coefficients in the RSG task is unique to that task, and reflects differences in the individual strategies of pseudo-random number generation.

## Discussion

### Summary of Results

We studied the cognitive mechanisms underlying the generation of random sequences by human participants. We found that a valuation-system approach, modeled using a logistic regression function, can qualitatively account for several previously-reported deviations from randomness and can be used to quantitatively predict the next generated symbol in the sequence. This approach also allowed us to quantify sequential effect in this task and compare them to an OL task. We found that sequential effects in the RSG task are substantially smaller than those in the OL task. Surprisingly, the heterogeneity in the sequential effects in the RSG task was substantially larger than that of the OL task. These results suggest that the RSG task is performed by a heterogeneous suppression of sequential effects.

### Heterogeneity: Random Sequence Generation vs. Operant Learning

The fact that there are differences in the behaviors of different people is obvious to even the most casual observer [39]. However, the extent that this contribution is important for understanding the cognitive strategies adopted by the different participants remains an open question. Here we found that in the RSG task, considering heterogeneity has proven to be essential both for predicting behavior and for understanding the seemingly non-monotonous sequential effect. By contrast, heterogeneity of sequential effects in an OL task is substantially smaller. This result is consistent with our previous study, in which we found that when predicting human preference in an OL task, the contribution of the heterogeneity to the predictive power is small [40].

It should be noted that the heterogeneity between participants in the RSG task is conceptually different from between-participants’ heterogeneities in other sequential tasks. In other tasks, the participant interacts or responds to external stimuli. For example, in an OL task, participants change their choice behavior in response to the sequence of past reinforcements. Conceptually, the behavior of the participant is a function from the sequence of external stimuli to a generated sequence of actions. This function is characterized by parameters such as the learning rate, which can be considered as a “traits” (although substantial heterogeneities between days in the same subject have also been reported [41]). Indeed, heterogeneity between participants in the learning rate, has been observed in the laboratory [40] as well as in natural conditions [42]. By contrast, the generation of random sequences is autonomous and should not be a function of external input. If done correctly, the RSG task is parameter-free and therefore we expect similarity in the behavior of the participants. Nevertheless, we find that rather than being “random”, people behave as if they utilize their past own actions as inputs to the “random” sequence generation, resulting in sequential dependencies. These sequential dependencies vary between participants, resulting in substantial heterogeneity in behavior. These results suggest that the RSG task involves active suppression of sequential dependencies, and that the cognitive strategies underlying this suppression differ between the different participants. Further studies should investigate the stability of these strategies over time spans larger than the one analyzed in the current experiment.

### Learning to be Stochastic

Previous studies have demonstrated that stochasticity can be learned by feedback in humans and in animals [9,43]. In our framework, such learning would correspond to decreasing the absolute value of sequential coefficients *β*_*l*_ in Eq (3). Thus, one potential interpretation to the heterogeneity in these coefficients is that it results from heterogeneities in this learning process, prior to the experiment.

### Valuation System

When studying OL, a dominant view is that the values in Eq (3) are not merely a phenomenological description of behavior. Rather, the reward-dependent components of Δ*Q* are explicitly represented by the activities of single neurons in the brain [32,33] (see [34] and [35], for review). Intriguingly, sequential effects in Eq (3) are comparable to these values, bringing up the possibility that in those OL tasks, the neural activity of “value” neurons also incorporate the sequential effects as in Eq (3). Experimentally, this hypothesis implies that the activity of those “value” neurons is also correlated with past actions, independently of the resultant rewards. Taking this possibility one-step further, these “value” neurons may also reflect the sequential effects of the RSG task. More generally, these results are consistent with the hypothesis that choice preferences in the brain are encoded through the valuation system, even when the choice outcome is not associated with primary rewards [44,45].

It will be difficult to test this hypothesis for the RSG task using monkey electrophysiology because of the obvious difficulty of instructing non-human primates to generate random sequences (but see [43]). However, it may be possible to do it by comparing brain activities of humans in OL and RSG tasks using functional Magnetic Resonance Imaging (fMRI).

### Predictability of Behavior

In this study we demonstrated that it is possible to correctly predict approximately 60% of a sequence in a RSG task based on previous choices. To what extent can we improve our predictions by using more data? Our stationary analysis indicates that the participants’ parameters are stationary at the time-scale of the experiment. These results suggest that the predictive power of the model is expected to increase if longer sequences are used. Nevertheless, when testing the predictive power of the model as a function of the training set size we find that performance saturates at approximately 500 trials (not shown), indicating that the improvement associated with a larger training set will not be substantial.

Can we make better predictions by using more sophisticated models? To address this question, we used an alternative nearest-neighbor model (under a Hamming distance function) in order to predict behavior. This model is approximately optimal in the limit of an infinite number of samples and stationarity. This model does not significantly outperform the logistic regression model (Fig 4A). This illustrates that the logistic model, at least in its heterogonous version, is close to the optimal non-parametric methods, albeit with a smaller number of parameters.

What is the source of the residual, unexplained stochasticity? One possibility is that it reflects irrelevant external inputs, not accounted for by the experimentalists. Another possibility is that this stochasticity reflects the macroscopic dynamics of the brain, e.g., a complex cognitive strategy such as a strategy that implements chaotic dynamics. A third alternative is that the unexplained stochasticity reflects trial-to-trial variability in the activities of individual neurons, and as such, amplifies microscopic variability. It is not clear how to experimentally dissociate these two possibilities and addressing this question awaits future studies [7].

### Randomness Cost

A recent theory has suggested that random choice behavior results from computational limitations on the policy [46]. In this framework, optimal policy is assumed to be a tradeoff between expected reward and expected information cost. Information-wise, a stochastic policy is preferable to a deterministic one. Therefore, everything else being equal, the agent should behave randomly. Our results suggest that by contrast to that assumption that randomness is information-wise cheap and thus cognitively easy, a stochastic policy is a cognitive challenge for human participants.

### Methodological Note: Insight versus Prediction

The predictive powers of the heterogenic version of the logistic model, the Levenshtein model and the NN-hamming model are comparable. However, we believe that for the study of cognitive processes in sequential tasks, the logistic model is more useful than the two latter models. The Levenshtein and the NN-hamming models are pattern based. They make predictions by seeking the previous outcomes of “similar” sequences. By contrast, the logistic model is restricted to simple linear non-linear dependencies. The advantage of the pattern-based approach is that given sufficient data, it can learn very complex strategies in stationary sequences. However, this lack of restrictions in the pattern-based approach results in models that are characterized by a large number of parameters (in the limit of a large dataset, 2^*L*^ parameters in the pattern-based models vs. *L* parameters in the logistic regression model) and complex dependencies that are very difficult to interpret in terms of a cognitive strategy. In this study, the logistic regression model enabled us to sheds light on the sequential effects and the nature of the variability between participants at no cost with respect to the predictive power of the model.

## Materials and Methods

### Experimental Design

#### The random sequence generation task

The experiment was carried by [15] and the full details of the task, participants and protocols appear in the original publication [15]. In short, 30 undergraduate students, who participated for course credit and received no payment, were asked to imagine that 100 people had tossed a coin, each 10 times, and the results had been recorded in a table of 100 rows and 10 columns, with each row recording the outcomes of the 10 tosses by 1 person. They were asked to produce a table of the same size in such a way that if it were compared with the one that represented actual coin tosses, it would not be possible to tell with statistical tests which table represented the actual coin tosses and which did not. We treated each participant’s sequence as a stream of 1,000 choices.

#### The operant learning task

The experiment was carried by [31] and the full details of the task, participants and protocols appear in the original publication [31]. In short, 200 participants were instructed to repeatedly choose between two unmarked alternatives in blocks of 100 trials. In each trial, pressing a button resulted in the delivery of a monetary payoff. Different blocks (problems) differed in reward schedule parameters. Each participant was tested in 12 problem sets. In total there were, 120 different problems, 2400 blocks and 240,000 trials.

### Data Analysis

Data analysis was conducted using MATLAB® (version 8.3) and statistical functions of the Statistics Toolbox (version 9.0). Of special importance for the paper were the functions for logistic regression model estimation and evaluation (*glmfit* and *glmval*, respectively). Both functions were used with default parameters. Note that these function assume independence between samples conditioned on the predictors (past action). This should not be confused with the dependency of the sequence itself and therefore the methods are valid.

For the heterogeneous models, prediction error rates were estimated by a ten-fold cross validation and averaged across folds and participants. For the homogenous models, we used a variation of a “leave-one-out” cross validation scheme to estimate these rates, where each participant data was predicted by training on other participants.

For the discounting model, according to which recent trials received higher weight (see Stationary Analysis), we used the *glmfit* with a vector of prior weights, which are equivalent to the inverses of the relative variances of the different observations (see *glmfit* documentation for more details). Testing started from the middle sample (501) for each participant, where training utilized all preceding trials. The exponential weight given for sample *k* trials ago was (1–*η*)·*η*^*k*^, and the values of *η* that were tested were zero and 25 non-zero values, between 10^−6^ and 1, evenly distributed on a logarithmic scale.
